# Simulation based comparison between a transversal and a tangential memristor model with a capacitance in parallel

**DOI:** 10.1371/journal.pone.0221533

**Published:** 2019-08-23

**Authors:** Oliver Pabst, Ørjan Grøttem Martinsen

**Affiliations:** 1 Department of Physics, University of Oslo, Oslo, Norway; 2 Department of Clinical and Biomedical Engineering Oslo University Hospital, Oslo, Norway; Institut Jean Lamour, FRANCE

## Abstract

In non-linear measurements, the applied stimulus itself affects the electrical properties of the underlying tissue. If corresponding voltage-current plots exhibit pinched hysteresis loops with pinched point in the origin of coordinates, the tissue can be classified as a memristor. Several organic memristors like human skin, venus flytrap and slime mould memristors have been demonstrated. However, measurements on organic memristors are usually affected by parasitic elements like a capacitance which will influence the appearance of the recorded pinched hysteresis loops. Here we study the parallel connection of two different memristor types, one with tangential and the other with transversal pinched hysteresis loop, and a capacitance by simulations. The simulations are inspired by human skin; beside the sweat ducts that can be modelled as a transversal memristor, the surrounding tissue, the stratum corneum exhibits non-linear electrical properties, as well. Based on a systematic study we suggested that the stratum corneum may be modelled as a tangential memristor. We demonstrate here by simulations that hysteresis loops with two pinched points can be achieved if a tangential memristor model is connected in parallel to a capacitance. Similar results were obtained from the skin recordings of some subjects; examples are presented here. Furthermore, if both the tangential and the transversal memristor models contribute to the simulation, quite asymmetric pinched hysteresis loops are obtained which are similar to recordings of some other test subjects.

## Introduction

**Fig 1:**
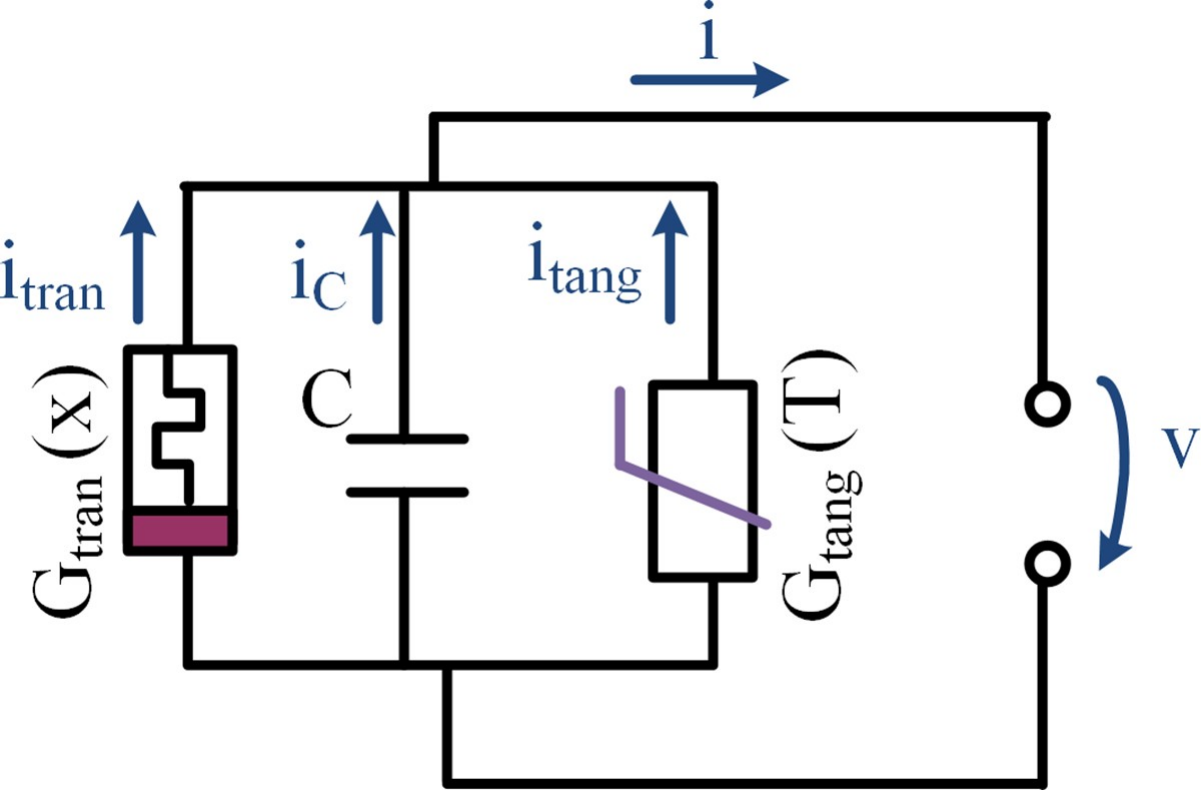
Electrical circuit model that is basis for the simulations. It is related to the non-linear electrical circuit model of human skin presented in [5]. *G*_*tran*_(*x*) represents a memristor model that exhibits transversal pinched hysteresis loop. Here the (modified) Hewlett Packard (HP) memristor model [6] is used and the inner state *x* describes the extension of the doped layer. *G*_*tang*_(*T*) is a memristor model that exhibits tangential pinched hysteresis loop. In this model the memductance dependents on the temperature *T*.

The memristor was found as the missing link between charge and flux and is labelled as the fourth passive circuit element [1]. It can be expressed as a state dependent resistor (“**mem**ory **r**es**istor**” **→ memristor**) that connects voltage and current via the state dependent Ohms law. The resulting voltage current (V-I) plot exhibits pinched hysteresis loop with pinched point in the origin of coordinates (fingerprint of a memristor [2]). Different memristor classes (ideal, ideal generic, generic, extended) are defined [3] and a generic memristor, for example, can be described by

v=M(x)i (1)

$$\frac{dx}{dt}=f\left(x,i\right)$$ (2)

using memristance *M*(***x***) (in analogy to resistance) where ***x*** is a vector of state variables. In a parallel connection, the description in terms of memductance (see Fig 1) (non-linear, state-dependent analog of conductance *G*) instead of memristance is useful. Beside different memristor classes [3], memristors are also divided by whether the two branches of the loop cross the origin of coordinates with different slopes (“transversal”) or touching it with equal slopes (“tangential”) [4]. The former shall be referred here as “transversal memristor” and the latter as “tangential memristor”.

The research on memristors became popular after a first realization (based on titanium dioxide) was presented in 2008 [6]. Memristors based on different materials, such as tantalum oxide [7, 8], zinc oxide [9] have been shown and applications of memristors can be found, for example, in neuromorphic computing [10–12] or in circuits emulating arithmetic operations [13, 14]. However, biological memristors (organic memristors), such as human skin [5, 15, 16], the venus flytrap [17] and slime mold memristors [18], have also been demonstrated and are based on intrinsic non-linear electrical properties. An electrical measurement that identifies these properties is non-linear in that sense, that the applied stimulus itself affects these properties and consequently the measurement.

Measurements on organic memristors are usually affected by parasitic elements [2, 19] that can cause a shift in the pinched point position away from the origin of coordinates. Several simulations based on two different (tangential) memristor models and different parasitic elements are demonstrated in [19]. A capacitance in parallel to a transversal memristor causes a shift in the pinched point position away from the origin of coordinates [20].

Here we study the voltage current characteristics of a memristor model that exhibits transversal pinched hysteresis loop in comparison to a memristor with tangential pinched hysteresis loop. We are especially interested in the connection of both types in parallel to each other and the parallel connections of both types with a capacitance. The simulations here are inspired by the non-linear electrical properties of human skin. Based on data presented in [16], Johnson et. al (2011) concluded that the sweat ducts in human skin can be modelled as a memristor [15]. This memristor exhibits a transversal pinched hysteresis loop. If the applied electrical stimulus is strong enough, electro-osmosis, the directed emotion of liquids, will occur within the sweat ducts. Dependent on the polarity of the applied voltage the sweat is pulled towards the skin surface or is pushed inwards towards deeper skin layers. The state dependent conductance increases in the former and decreases in the latter case.

**Fig 2:**
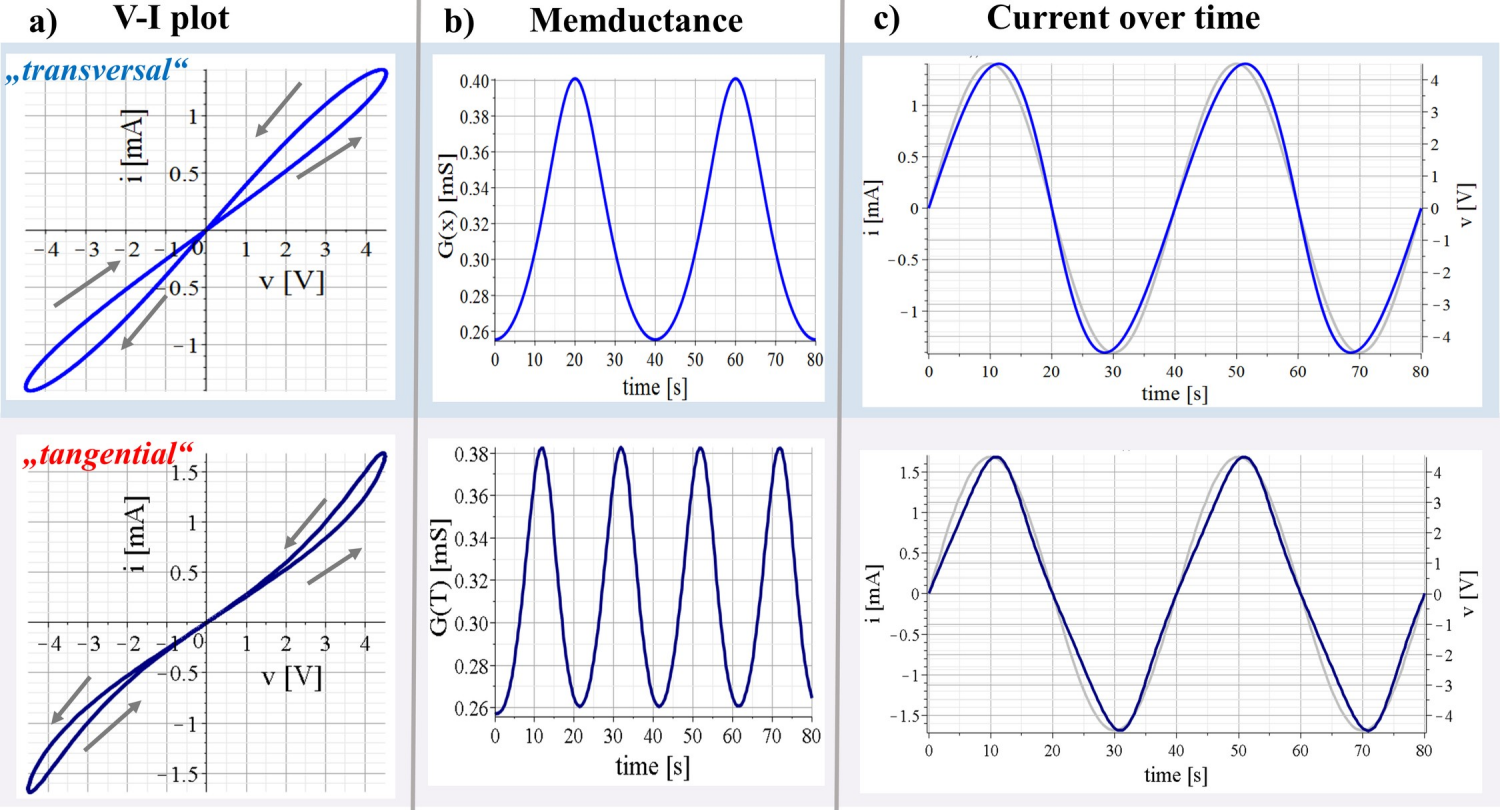
Results of simulations on two different memristor types. A sinusoidal voltage, *v*, with amplitude of 4.5 V and frequency of 0.025 Hz was used as signal source in all simulations. All results are shown over two periods. The results of the (modified) transversal HP memristor model (Eqs (7), (9) and (10)) are shown in the upper line. The results of the tangential memristor model (Eqs (5) and (6)) are presented in the lower line. (a) Voltage current (V-I) plots. The two branches of the hysteresis loop in the upper plot are crossing the pinched point with different slopes (transversal) and touching with equal slopes in the lower plot (tangential). The arrows indicate the orientation of the hysteresis loops. (b) Corresponding memductance changes over time of both memristor types. (c) Applied voltage and corresponding currents over time.

A second type of non-linear electrical properties was reported by Panescu et. al [21]; The conductance of the stratum corneum increases with an increase in temperature. The stratum corneum consists of keratinized tissue [22] like human hair that also shows a noticeably conductance increase with increasing temperature [23]. Independent of the sign of the applied stimulus, if a current passes the stratum it increases its temperature and consequently the conductance. The stratum corneum may be modelled as a memristor itself [5] and the increase in conductance independent of the sign of the applied stimulus may be an indication for a tangential memristor (see Fig 2).

The transversal sweat duct memristor and the tangential stratum corneum memristor are electrically in parallel to each other within the measurements [5]. The stratum corneum exhibits capacitive properties that are related to its humidity content [24] that are electrically in parallel to both memristor types.

In a systematic study on 28 test subjects (see [5]) it was found that the sweat duct memristor usually dominates the measurement as long as good galvanic contact through the sweat ducts was given. However, if the galvanic contact was little, the current travelled mainly through the stratum corneum.

## Methods

All simulations were done by the use of Maple (Version 2016.2). Obtained differential equations were solved numerically by the Runge-Kutta-Fehlberg 45 method. A constant amplitude sinusoidal voltage source is used in all simulations.

### Memristor model with a tangential pinched hysteresis loop

The here chosen tangential memristor model is presented in [19]. It is basically a negative temperature coefficient (NTC) thermistor model that exhibits pinched hysteresis loops in the voltage-current representation for different amplitudes and excitation waveforms. Thus, it is a memristor model, as well [2, 19] and it is described as follows:

$$G\left(T\right)={\left(R_{0N}\;e^{\beta_{N}\left(\frac{1}{T}-\frac{1}{T_{0N}}\right)}\right)}^{-1}$$ (3)

$$\frac{dT}{dt}=\frac{\delta_{N}}{H_{CN}}\left(T_{0N}-T\right)+\frac{G\left(T\right)}{H_{CN}}v^{2},$$ (4)

where *G*(*T*) is the temperature-dependent conductance; *R*_*ON*_ = 3.89 kΩ is the resistance at ambient temperature *T*_*ON*_ = 300 K; *β*_*N*_ = 5 × 10^5^ K is the material-specific constant; *δ*_*N*_ = 0.1 W/K is the dissipation constant; and *H*_*CN*_ = 0.14 J/K is the heat capacitance. Simulation results of this model are shown in the lower line of Fig 2. An initial state of *T = T*_*ON*_ = 300 K was used for the simulations here.

To distinguish between the temperature, *T*, and the time of one full period of an applied signal, the latter shall be referred as *t*_*period*_ within this paper.

### Memristor model with a transversal pinched hysteresis loop

The Hewlett Packard (HP) memristor [6] is described by the equations

$$M\left(x\right)=R_{on}\cdot x+R_{off}\cdot \left(1-x\right)$$ (5)

$$\frac{dx}{dt}=\frac{\mu_{v}\cdot R_{o}n}{D^{2}}\cdot i_{tran}(t),$$ (6)

with *x* as the internal state variable which reflects the extension of the high conductive (doped) region, normalized with the absolute extent *D* of the titanium dioxide layer which is equal to 10 nm (in [6], the internal state was given as the total extension *w* of the doped region instead of the normalized extension *x*). The state *x* can obtain values between 0 to 1. If *x* is equal 1, the overall memristance is lowest and equal to *R*_*on*_ = 0.1 kΩ, while a state *x* = 0 reflects the highest memristance with M(x) equal to *R*_*off*_ = 16 kΩ. An initial state of *x* = 0.76 was used for every simulation within this paper. The constant *μ*_*v*_ = 10^−14^ m^2^/(sV) reflects the oxygen vacancy mobility.

In a parallel circuit, the expression as state-dependent

$$G_{tran}(x)=\frac{1}{M(x)}$$ (7)

conductance is useful.

The inner state, *x*, of the HP memristor model changes much faster than that of the NTC thermistor model and would reach its boundaries almost instantly for sinusoidal voltage source with amplitude of 4.5 V and *f* = 0.025 Hz (example voltage that is used for the simulations). Therefore a modification of the state equation (see Eq (8)), is used here and given as

$$\frac{\mathrm{dx}}{\mathrm{dt}}=\frac{\mu_{v}\cdot R_{\mathrm{on}}}{D^{2}}\cdot i_{tran}\left(t\right)\cdot 0.0005.$$ (8)

The factor of 0.0005 slows the state change of *x* down. Simulation results from this (modified) HP memristor model are presented in the upper line of Fig 2.

### Resulting currents

The electrical circuit model in Fig 1 represents all three elements (transversal memristor with *G*_*tran*_(*x*), tangential memristor with *G*_*tang*_(*T*) and capacitance *C*) that are used for the simulations within this paper.

The corresponding currents are described as follows

$$i_{C}=C\frac{dv}{dt}$$ (9)

$$i_{tang}=vG_{tang}\left(T\right)$$ (10)

$$i_{tran}=vG_{tran}(x).$$ (11)

The overall current, *i*, of the parallel circuit in Fig 1 can be expressed by

$$i=i_{tran}+i_{tang}+i_{C}$$ (12)

and the overall state dependent admittance around an equilibrium state (*x*_*Q*_, *T*_*Q*_) is expressed by

Y(x,T)=G(x,T)+jB (13)

$$=G_{tran}(x)+G_{tang}(T)+j\omega C$$ (14)

with a susceptance of *B*, an imaginary unit of *j* and *ω* = *2πf*.

**Fig 3:**
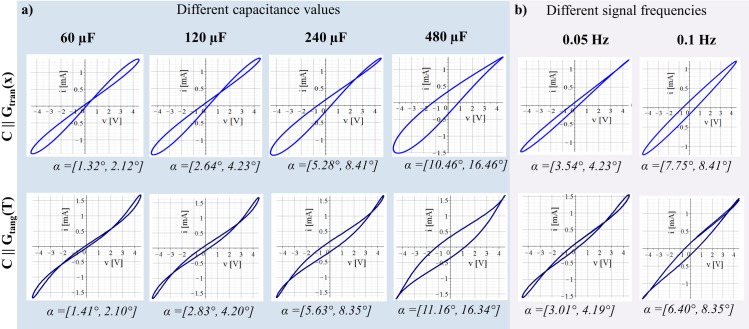
V-I plots obtained from simulations of the single memristor models with a capacitance in parallel, all carried out with applied sinusoidal voltage *v* with amplitude of 4.5 V. Simulations were carried out over two signal periods. The upper plots represent the simulations of the transversal HP memristor model with a capacitance in parallel (*i* = *i*_*tran*_+*i*_*C*_) and the lower plots represent the simulations of the tangential memristor (NTC thermistor) model with a capacitance in parallel (*i* = *i*_*tang*_+*i*_*C*_). The range of the phase shift *α* between the voltage and the current is given under each plot. (a) Different capacitance values as indicated. The signal frequency *f* of the applied voltage was 0.025 Hz. (b) Different signal frequencies of the applied voltage as indicated. The capacitance value *C* was 60 μF.

**Fig 4:**
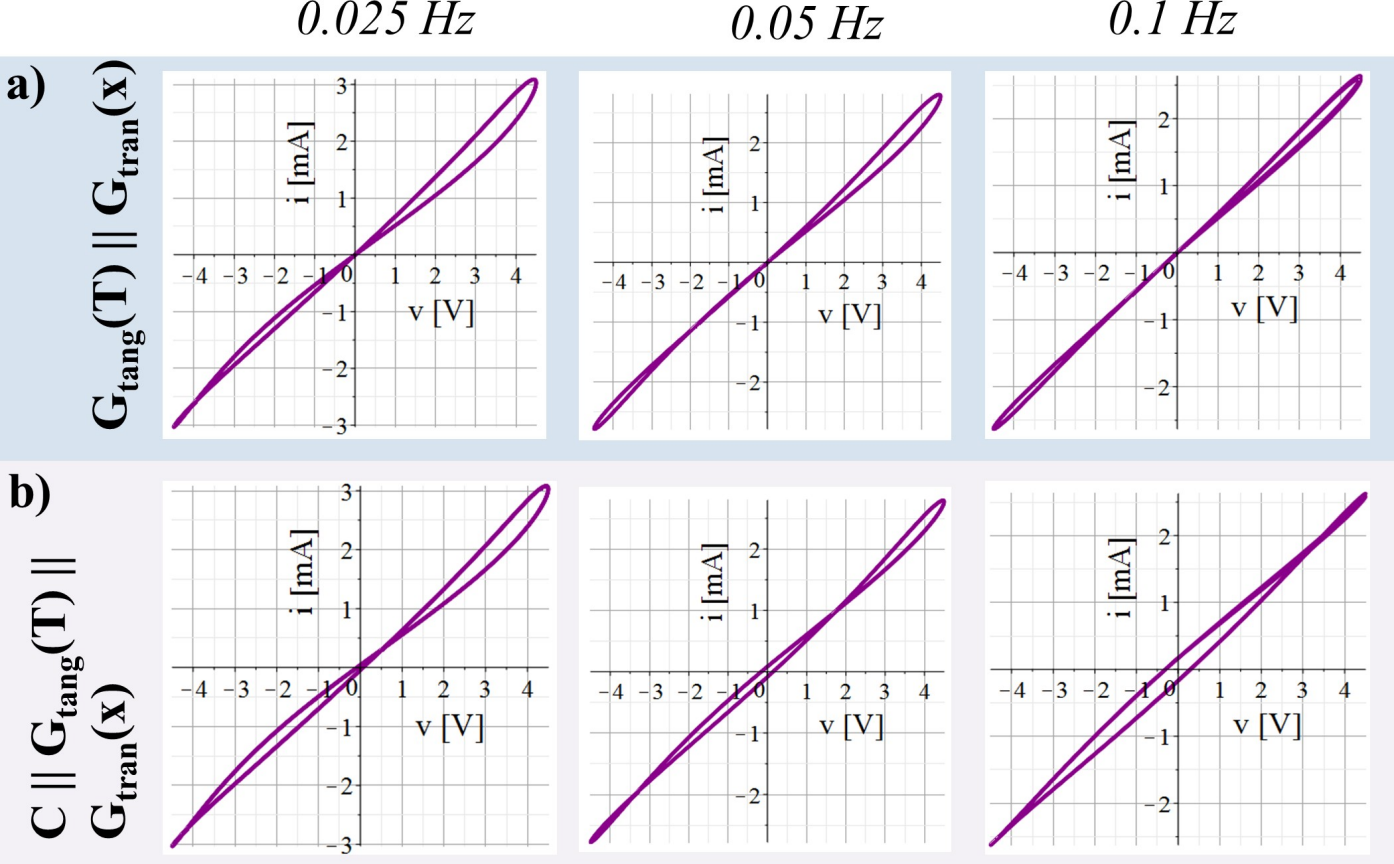
V-I plots obtained from simulations of both memristor models connected in parallel. The simulation is carried out for different signal frequencies of applied sinusoidal voltage *v* with amplitude of 4.5 V. Simulations were carried out over two signal periods. (a) Both memristor models only in parallel to each other. (b) Capacitance in parallel in addition to the two memristor models as it is shown in Fig 1.

Results of the parallel connections of only two of the three elements are shown in Figs 3 and 4A. The results of all three elements in parallel are presented in Fig 4B.

### Calculation of phase angles

A capacitance in parallel to a memristor will cause a phase shift between the voltage and the current that can be expressed by

$$\alpha \left(x\right)=\tan^{-1}\frac{2\pi fC}{G_{tran}\left(x\right)},$$ (15)

with *α*(*x*) as a function of the extension of the doped layer in case the of transversal HP memristor model and by

$$\alpha \left(T\right)=\tan^{-1}\frac{2\pi fC}{G_{tang}\left(T\right)},$$ (16)

with *α*(*T*) as a function temperature in case the of the here chosen tangential memristor model (NTC thermistor). Since the phase shift is a function of the inner state in both cases, it changes during one period. The maximum and minimum values that the phase angle can obtain during one period was calculated by the minimum and maximum state-dependent conductance values that were achieved during this period.

## Results and discussion

### Simulations of the single memristor models

Essential for any memristor is the pinched hysteresis loop in the V-I plot with pinched point in the origin of coordinates as both memristor models exhibit (Fig 2A). However, in case of the (modified) HP memristor model, the two branches of the hysteresis loop are crossing the pinched point with different slopes which makes it a transversal memristor. The two branches of the hysteresis loop in the tangential memristor model are touching the pinched point with equal slopes. The change of the inner state *x* in the HP memristor model depends on the sign of the electrical signal (current *i* in this case). As long as the applied voltage and the corresponding current, *i*, are positive, the memductance increases (Fig 2B) and the memductance maximum is reached after the positive halve of the period. The memductance decreases as long as the applied voltage is negative and the memductance minimum is achieved after the negative halve of the period. The memductance exhibits consequently one maximum state during one period.

In contrast, the change of the internal state *(T*) of the here used tangential memristor model is a function of *v*^*2*^ (see Eq 6). Thus, it is independent of the sign of the applied voltage. As the temperature, *T*, increases with the applied voltage, the summand $\frac{\delta_{N}}{H_{CN}}\left(T_{0N}-T_{N}\right)$ in Eq (6) causes a decrease in temperature that counteracts. As a consequence, two memductance maxima occur (see Fig 2B) during one period which is different from the HP memristor model. These maxima are close to ¼ t_period_ and ¾ t_period_, which is the reason why the obtained maximum current is larger than it was for the HP memristor model. The orientation of the pinched hysteresis loop in the first quadrant is counter clock-wise for both models (Fig 2A). Within the third quadrant, the orientation of the transversal HP memristor model is clockwise while it is counter-clockwise for the here used tangential memristor model.

Within the simulations on the single memristor models, there are no parasitic elements (like a capacitance) that cause any phase shift. However, the peaks of the obtained currents are not at *t*_*period*_ /4 (at 10s in the simulations here with 0.025 Hz) which is different from the sinusoidal voltage source (Fig 2C). This is because of the change in the state-dependent conductance. As the sinusoidal voltage starts to drop slightly after *t*_*period*_ /4, the state dependent conductance of both memristor models is still increasing. As long as the impact of the state change on the resulting current is larger than the voltage drop, the current increases further, and its maximum is behind the voltage maximum. Differences between both memristor models can be observed during the negative halve. The state-dependent conductance of the here used tangential memristor model (NTC thermistor) increases during the negative halve, as well, and the current minimum is behind the voltage minimum. However, since the negative voltage causes a decrease in the state-dependent conductance of the transversal HP memristor model, the minimum of the corresponding current is ahead of the voltage minimum.

The sign of the voltage source amplitude will affect the results of the transversal HP memristor model (compare upper lines in Fig 2 and S1 Fig). If the sign is negative (S1 Fig), the memductance decreases in the first halve of the period and increases back to the initial value in the second halve of the period. The results of the here used tangential memristor model are independent of the sign of the voltage source. The simulation results in Fig 2A and 2B are similar to those in S1A and S1B Fig.

If a DC offset is applied to the voltage source, the transversal HP memristor exhibits a cumulative state change among the periods and the resulting pinched hysteresis loop changes from period to period. This is different for the tangential memristor; a DC offset will not cause a cumulative state change (see S2 Fig).

### Simulations of the single memristor models with a capacitance in parallel

The phase angle *α*(*x*) decreases (since the state dependent conductance increases) within the positive halve of the voltage and decrease in within the negative halve. The phase angle *α*(*T*) decreases within the first quart, increases within the second quart, decreases within the third quart, and increases again within the fourth quart of the applied sinusoidal voltage.

The range of *α* in each simulation is given under the pots in Fig 3.

In the V-I plots, a capacitance in parallel to the transversal HP memristor will cause a shift in the pinched point position away from the origin of coordinates (Fig 3A, upper line). As the capacitance value increases the pinched point shifts away from the origin of coordinates. However, a capacitance in parallel to the transversal HP memristor model will not cause an additional pinched point.

Hysteresis loops with two pinched points (that are symmetric with regard to the coordinate origin) were obtained by the simulation if the capacitance is connected in parallel to the tangential memristor model (Fig 3A, lower line). With an increase in the capacitance value, both pinched points move symmetrically away from the origin of coordinates (symmetric with respect to the origin of coordinates).

In Fig 3B) the simulations were done for different signal frequencies but a constant capacitance value of 60 μF.

The susceptance (*B* = 2*πfC*) in the simulation with 0.05 Hz and 60 μF is the same as it is for the simulation with 0.025 Hz and 120 μF (see Fig 3A). However, the higher the frequency the less state change happens in the corresponding memristors which is the reason for the difference in the V-I plots in both cases. The same applies for the example with *C* = 240 μF and *f* = 0.025 Hz in comparison to the example with *C* = 60 μF and *f* = 0.1 Hz.

### Simulations of both memristor models in parallel

The parallel connection of both different memristor models exhibits hysteresis loops with pinched point in the origin of coordinates (Fig 4A). It can therefore be modelled as a memristor itself which is in accordance with the closure theorem [1].

The obtained pinched hysteresis loops in Fig 4A are quite asymmetric. The width of the lobe in the third quadrant is much smaller than that of the first quadrant. From 0/4 *t*_*period*_ to ¼ *t*_*period*_ (of sinusoidal voltage with positive sign of the amplitude) both memristor types exhibit an increase of the state dependent conductance and the increase in current is large. From ¼ *t*_*period*_ to 2/4 *t*_*period*_, the state-dependent conductance of the here used tangential memristor model decreases while it still increases for the transversal HP memristor model (both are counteracting). The resulting change in the overall state dependent conductance is relative small. From 2/4 *t*_*period*_ to ¾ *t*_*period*_, the state dependent conductance of the here used tangential memristor model increases, while it decreases for the transversal HP memristor model and the overall state change is small. From ¾ *t*_*period*_ to 4/4 *t*_*period*_ both memristors exhibit a decrease in in the state dependent conductance.

The parallel connection of both memristor types and a capacitance (as illustrated in Fig 1) results in asymmetric pinched hysteresis loops with two pinched points (Fig 4B).

### Relation between the simulations and human skin

The chosen memristor models for the simulations here are not related to the physiological properties of human skin; and the used models exhibit for example much higher conductance values. The conductance value of around 260 μS at simulation start (see Fig 2B) is not specific and originates basically from the chosen tangential memristor model as it is given in [19]. For the comparison between both models, the internal state, *x*, of the transversal HP memristor was chosen to be 0.76 at simulation start to achieve a similar conductance value. The conductance of human skin in [5], was several μS up to several tens of μS. However, there are some similarities between the chosen memristor models and the non-linear electrical properties of human skin. The tangential memristor (NTC thermistor) model that is chosen here exhibits an increase in the state dependent conductance with temperature increase like the stratum corneum [21]. The inner states of the HP memristor and the sweat duct memristor can both be described by the extension *x* of the high conductive region vs. the low conductive region. The HP memristor (model) can be classified as an ideal generic memristor [2] and is different from the sweat duct memristor, which is a generic memristor up to a certain magnitude of applied voltage [5]. Anyway, important for the choice of the memristor models here is that one of the models exhibits a tangential hysteresis while the other one exhibits a transversal pinched hysteresis. Example recordings from human skin that represent three different cases, one in which the sweat duct memristor dominates the measurement, one in which the stratum corneum memristor dominates the measurement, and one in which both memristors noticeably contribute to the measurement, are presented in S3 Fig. The susceptance (imaginary part of the admittance) of human skin at low frequencies is mainly determined by the capacitive properties of the stratum corneum that are related to its humidity [24]. Sweat that reaches the skin surface diffuses partly into the stratum corneum and can change its capacitive properties. However, these changes are quite small and slow compared to changes in the conductance. Therefore, we chose to use a constant capacitance (not state-dependent) for the simulations. The effect of different (constant) capacitance values on the pinched hysteresis loops is shown in Fig 3. Further research needs to be done to clarify whether the capacitive component in the stratum corneum can be directly affected by the applied voltage itself, and thus might be modeled as a state dependent component. To reflect the findings from [5], the use of a constant capacitance in the simulations here (see Fig 1) was sufficient. Since the simulations are done at very low frequencies (e.g. f = 0.025 Hz) and the state dependent conductance values of the memristor models are quite high, only large capacitance values of 60 μF and above showed noticeably effects on the pinched hysteresis loops. These capacitance values are just chosen with regard to the chosen memristor models and do not relate to the capacitance values of human skin, for which several tens of nF were recorded (see [5]).

## Conclusions

A comparison between a tangential and a transversal memristor model was done by simulations using a sinusoidal voltage source. A capacitance in parallel to a transversal memristor will cause a shift of the pinched point away from the origin of coordinates. A hysteresis loop that is symmetric with respect to the origin of coordinates and that exhibited two pinched points was obtained from a tangential memristor model in parallel to a capacitance. Pinched hysteresis loops that were quite asymmetric (large lobe area in the first quadrant, small lobe area in the third quadrant) are obtained when both different memristor models (tangential, transversal) are connected in parallel to each other. The simulations here are inspired by human skin that can be modelled by a transversal memristor (non-linear properties originating from the sweat ducts) that is electrically in parallel to a tangential memristor (non-linear properties originating from the stratum corneum) and a capacitance (see [5]). Even though the sweat duct memristor often dominates the measurement as long as the galvanic contact through the sweat ducts is given [5], both memristor types can contribute noticeably to the measurement. Resulting pinched hysteresis loops in the voltage current plot are quite asymmetric similar to the simulation results of a tangential and a transversal memristor model in parallel (compare S3 Fig and Fig 4). However, the results from the simulations here may help understanding recordings from other biological tissues, as well. For example, when one observes symmetric hysteresis loops with two pinched points it is likely that the tissue can be modelled by a tangential memristor in parallel to a capacitance.

## Supporting information

S1 Fig Results of simulations on two different memristor types using a sinusoidal voltage with negative sign. A sinusoidal voltage *v* with amplitude of -4.5 V and frequency of 0.025 Hz was used as signal source in all simulations. All results are shown over two periods. The sign of the voltage amplitude has an effect on the results obtained from the transversal (adapted) HP memristor model (compare with upper line in Fig 2) while it does not for the here used tangential memristor (NTC thermistor) model. The results of the (modified) transversal HP memristor model (Eqs (7), (9) and (10)) are shown in the upper line. The results of the tangential memristor model (Eqs (5) and (6)) are presented in the lower line. (a) Voltage current (V-I) plots. The two branches of the hysteresis loop in the upper plot are crossing the pinched point with different slopes (transversal) and touching with equal slopes in the lower plot (tangential). The arrows indicate the orientation of the hysteresis loops. (b) Corresponding memductance changes over time of both memristor types. (c) Applied voltage and corresponding currents over time.
(TIF)

S2 Fig Results of simulations on two different memristor types using a sinusoidal voltage with a DC offset. A sinusoidal voltage, *v*, with amplitude of 4.5 V, frequency of 0.025 Hz and a DC offset of -0.25 V was used as signal source in all simulations. All results are shown over two periods. (a) V-I plot obtained from the (modified) transversal HP memristor model (Eqs (7), (9) and (10)). The appearance of the pinched hysteresis loop changes from period to period. (b) V-I plot obtained from the tangential memristor (NTC thermistor) model (Eqs (5) and (6)).
(TIF)

S3 Fig V-I plots obtained from real measurements on human skin. The presented results are examples from a systematic study on 28 test subjects (see (5) for additional recordings and further information). Each recording was done over three periods (green dotted plots represent the first period of each recording, red dotted plots the second period, and the solid plots represent the third period of each recording). The recordings were done by the use of a three electrode-system with dry Ag/AgCl measurement electrodes (active electrode area of 0.283 cm^2^). The orientation of the voltage is here from deeper skin layers to the skin surface, meaning that as the applied voltage is positive, the electrical potential at the skin surface is lower than that of the deeper skin layers. Here the results of applied sinusoidal voltage, *v*, with amplitude of 1.2 V and different signal frequencies of 0.1 Hz, 0.5 Hz, 1 Hz, and 2.5 Hz are shown. (a) Recordings from a subject K obtained from the forehead. This is an example, in which the transversal sweat duct memristor (in parallel to the capacitive properties of the stratum corneum) dominates the measurement. The hysteresis loop exhibits only one pinched point that shifts away from the origin of coordinates with increasing frequency (compare with the simulations presented in the upper line of Fig 3). (b) Recordings from subject E obtained from the earlobe. Hysteresis loops that are quite symmetric with regard to the origin of coordinates and that exhibit two pinched points can be observed. With increasing frequency both pinched points move away from the origin of coordinates. The recorded currents are quite small (below 5 μA for applied sinusoidal with 1.2 V amplitude) which is indication that the galvanic contact through the sweat ducts was little and the current went mainly through the stratum corneum. The recordings are therefore dominated by the tangential stratum corneum memristor (compare with the simulations presented in the lower line of Fig 3). (c) Recordings from a subject N obtained from the forehead. The pinched hysteresis loop recorded at 0.1 Hz is quite asymmetric with regard to the origin of coordinates and exhibits one pinched point. The lobe is much wider in the first quadrant than it is in the third quadrant as it was obtained by simulation in Fig 4A. The capacitive properties of the stratum corneum contribute noticeably in the recordings at 0.5 Hz, 1Hz and 2.5 Hz and the results can be compared with the simulations in Fig 4B. It is likely, that subject N represents a case in which both, the tangential stratum corneum, as well as, the transversal sweat duct memristor contribute noticeably to the measurements.
(TIF)
