# Supplementary figures and images for: Simulation based comparison between a transversal and a tangential memristor model with a capacitance in parallel
Source: PLoS One. 2019 Aug 23;14(8):e0221533. doi: 10.1371/journal.pone.0221533 (PMC6707609; doi:10.1371/journal.pone.0221533)

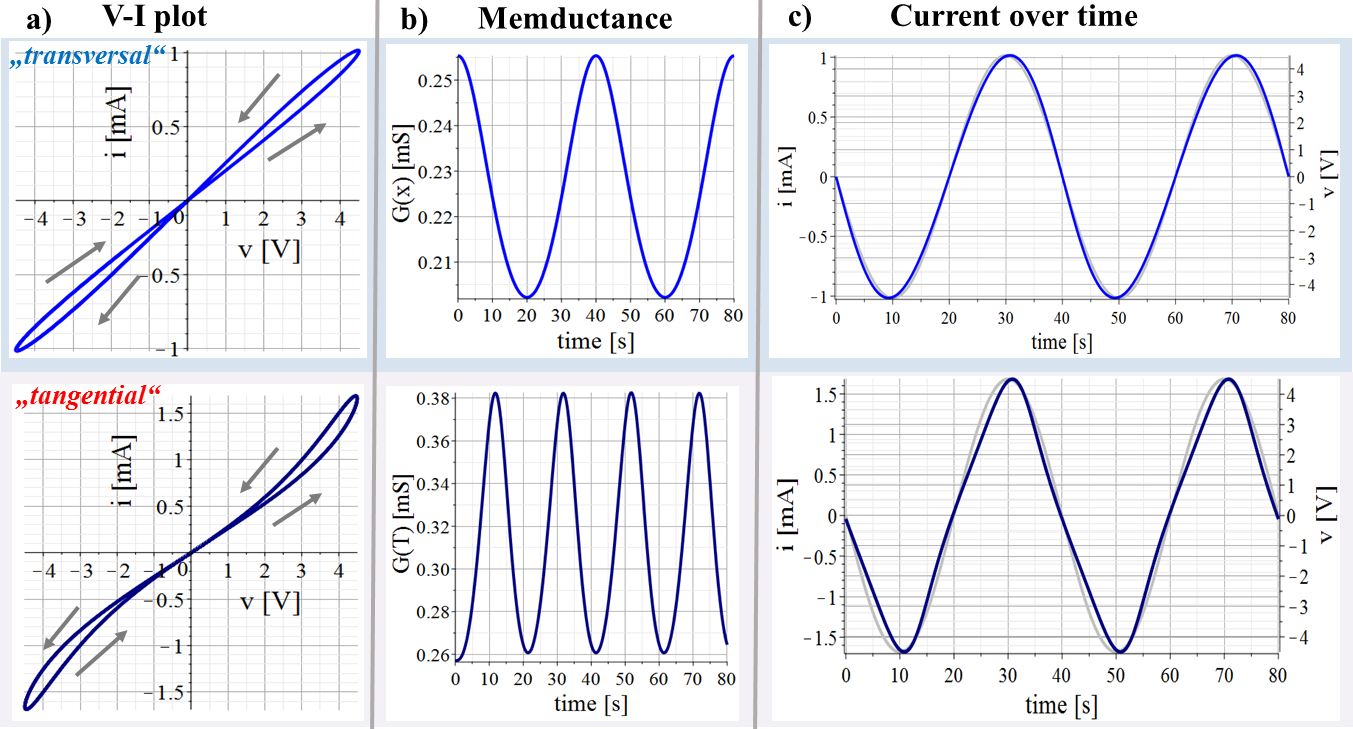

Supplement: S1 Fig — A sinusoidal voltage v with amplitude of -4.5 V and frequency of 0.025 Hz was used as signal source in all simulations. All results are shown over two periods. The sign of the voltage amplitude has an effect on the results obtained from the transversal (adapted) HP memristor model (compare with upper line in Fig 2) while it does not for the here used tangential memristor (NTC thermistor) model. The results of the (modified) transversal HP memristor model (Eqs (7), (9) and (10)) are shown in the upper line. The results of the tangential memristor model (Eqs (5) and (6)) are presented in the lower line. (a) Voltage current (V-I) plots. The two branches of the hysteresis loop in the upper plot are crossing the pinched point with different slopes (transversal) and touching with equal slopes in the lower plot (tangential). The arrows indicate the orientation of the hysteresis loops. (b) Corresponding memductance changes over time of both memristor types. (c) Applied voltage and corresponding currents over time. (TIF) [file pone.0221533.s001.tif]

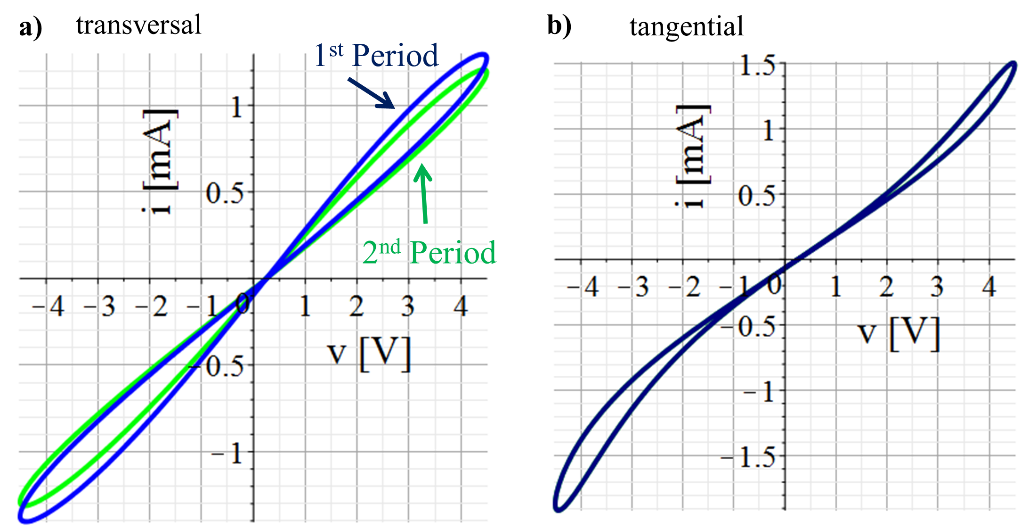

Supplement: S2 Fig — A sinusoidal voltage, v, with amplitude of 4.5 V, frequency of 0.025 Hz and a DC offset of -0.25 V was used as signal source in all simulations. All results are shown over two periods. (a) V-I plot obtained from the (modified) transversal HP memristor model (Eqs (7), (9) and (10)). The appearance of the pinched hysteresis loop changes from period to period. (b) V-I plot obtained from the tangential memristor (NTC thermistor) model (Eqs (5) and (6)). (TIF) [file pone.0221533.s002.tif]

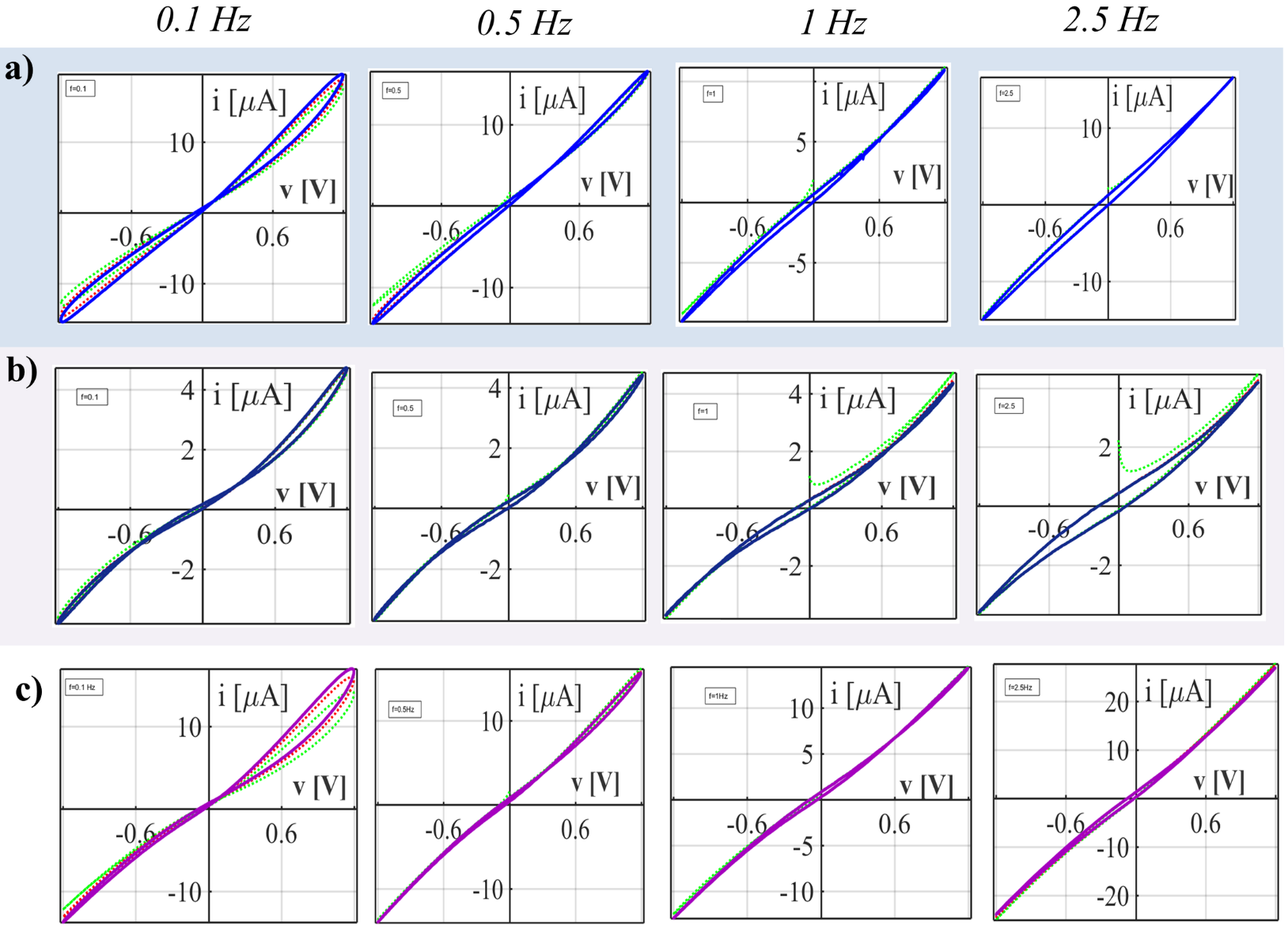

Supplement: S3 Fig — The presented results are examples from a systematic study on 28 test subjects (see (5) for additional recordings and further information). Each recording was done over three periods (green dotted plots represent the first period of each recording, red dotted plots the second period, and the solid plots represent the third period of each recording). The recordings were done by the use of a three electrode-system with dry Ag/AgCl measurement electrodes (active electrode area of 0.283 cm2). The orientation of the voltage is here from deeper skin layers to the skin surface, meaning that as the applied voltage is positive, the electrical potential at the skin surface is lower than that of the deeper skin layers. Here the results of applied sinusoidal voltage, v, with amplitude of 1.2 V and different signal frequencies of 0.1 Hz, 0.5 Hz, 1 Hz, and 2.5 Hz are shown. (a) Recordings from a subject K obtained from the forehead. This is an example, in which the transversal sweat duct memristor (in parallel to the capacitive properties of the stratum corneum) dominates the measurement. The hysteresis loop exhibits only one pinched point that shifts away from the origin of coordinates with increasing frequency (compare with the simulations presented in the upper line of Fig 3). (b) Recordings from subject E obtained from the earlobe. Hysteresis loops that are quite symmetric with regard to the origin of coordinates and that exhibit two pinched points can be observed. With increasing frequency both pinched points move away from the origin of coordinates. The recorded currents are quite small (below 5 μA for applied sinusoidal with 1.2 V amplitude) which is indication that the galvanic contact through the sweat ducts was little and the current went mainly through the stratum corneum. The recordings are therefore dominated by the tangential stratum corneum memristor (compare with the simulations presented in the lower line of Fig 3). (c) Recordings from a subject N obtained from t [file pone.0221533.s003.tif]
